# Overcoming P-Glycoprotein-Mediated Multidrug Resistance in Colorectal Cancer: Potential Reversal Agents among Herbal Medicines

**DOI:** 10.1155/2018/3412074

**Published:** 2018-08-12

**Authors:** Gayoung Lee, Jin-Yong Joung, Jung-Hyo Cho, Chang-Gue Son, Namhun Lee

**Affiliations:** ^1^Department of Clinical Oncology, Cheonan Korean Medicine Hospital of Daejeon University, 4, Notaesan-ro, Seobuk-gu, Cheonan-si, No. 31099, Republic of Korea; ^2^Liver and Immunology Research Center, Dunsan Korean Medicine Hospital of Daejeon University, 75, Daedeok-daero 176beon-gil, Seo-gu, Daejeon-si, No. 35235, Republic of Korea; ^3^Department of Internal Medicine, Graduated School of Korean Medicine, University of Daejeon, 62, Daehak-ro, Dong-gu, Daejeon-si, No. 34520, Republic of Korea

## Abstract

**Objectives:**

Multidrug resistance (MDR) is the major reason for the failure of chemotherapy in colorectal cancer (CRC), and the primary determinant of MDR in CRC patients is active drug efflux owing to overexpression of P-glycoprotein (P-gp) in cancer tissues. Despite research efforts to overcome P-gp-mediated drug efflux, the high toxicity of P-gp inhibitors has been a major obstacle for the clinical use of these agents. The aim of this study was to review the literature for potential P-gp reversal agents among traditional herbal medicines, which offer the advantages of safety and potential synergetic effects in CRC chemotherapy.

**Methods:**

We searched ten databases including 3 English databases, 1 Chinese medical database, and 6 Korean medical databases up to July 2018 and included in vivo and in vitro studies evaluating the effects of herbal medicines as P-gp reversal agents in CRC.

**Results:**

A total of 28 potentially related studies were identified and 16 articles were included. Involving 3 studies about* Salvia miltiorrhiza* and 2 studies about* Curcuma longa*, finally we found 14 kinds of traditional herbal medicines—*Salvia miltiorrhiza, Curcuma longa, Sinomenium acutum, Stephania tetrandra, Bufo gargarizans, Coptis japonica, Piper nigrum *and* Piper longum, Hedyotis diffusa, Schisandra chinensis, Glycyrrhiza glabra, Glycyrrhiza inflate, Daphne genkwa, Stemona tuberosa *Lour, and* Andrographis paniculata*—as showing efficacy as P-gp inhibitors in anticancer drug-resistant CRC cells in vitro and in vivo.

**Conclusions:**

This brief account provides insight into the relationship between P-gp and CRC. Further studies on herbal medicines with demonstrated effects against P-gp overexpression will aid in improving the efficacy of chemotherapy in CRC.

## 1. Introduction

Colorectal cancer (CRC) is the third most common cancer in the United States and over 50,000 deaths from CRC occurred in 2017 in the United States. [1]. Chemotherapy is regarded as an important treatment for CRC. However, the development of drug resistance usually results in the failure of chemotherapy in advanced CRC [2, 3]. Several mechanisms of resistance have been identified to date, including increased drug efflux, reduced drug intake, cell cycle disruptions, alterations in the drug target, and sequestration of drugs [4, 5]. In particular, ‘active drug efflux' by overexpressed ATP-binding cassette (ABC) transporters, which causes alterations in the intracellular accumulation of drug and thereby diminishes the efficacy of chemotherapy [6, 7], is cited as a common mechanism.

The P-glycoprotein (P-gp) transporter, referred to as ABCB1 (ATP-binding cassette subfamily B member 1) or MDR1 (multidrug resistance protein 1), is among the most clinically important ABC transporters in the context of the gastrointestinal system [3, 8]. P-gp is physiologically expressed in normal cells of the adrenal gland, pancreas, liver, brain, and intestines. In the human gastrointestinal tract, P-gp is highly concentrated on the apical surfaces of superficial columnar epithelial cells of the colon [9–11]. However, P-gp is pathologically associated with colorectal carcinogenesis and tumor grade [12, 13]. Since Juliano and Ling first discovered the role of P-gp in drug-resistant cells in 1976, the structure of P-gp has been extensively studied, with several models demonstrating the drug export mechanism of P-gp [14]. P-gp consists of a single polypeptide with two NBDs (nucleotide binding domains) and two TMDs (transmembrane domains), each of which contains six transmembrane helices (TMH). This structure allows the formation of a binding pocket, which determines the substrate specificity of P-gp [15].

Investigations of strategies for overcoming P-gp-mediated drug efflux during the last four decades have led to the development of several generations of P-gp inhibitors. However, the first three generations of P-gp inhibitors have shown disappointing results in humans because of the toxicity of these resistance-modifying agents [16]. Efforts to develop safer and more effective P-gp inhibitors have led researchers to consider plant-derived materials. Several researchers have demonstrated that alkaloids, flavonoids, and other plant compounds act as P-gp inhibitors when accompanied by chemotherapeutic drugs [17, 18]. Despite this large body of literature, no overviews of herbal medicines that act as P-gp reversal agents in CRC have been published. The present brief review has the goal of proposing the potential herbal medicines as P-gp reversal agents in CRC.

## 2. Methods

The following electronic databases were searched for studies from its inception to July 2018: English language databases (PubMed, EMBASE, and CINAHL), a Chinese literature database (China Academic Journal), and six Korean language databases (Korea Med, Oriental Medicine Advanced Medical Database (KM base), the Research Information Service System (RISS), National Digital Science Library, ProQuest Dissertations and Thesis, and Korean Studies Information Services System (KISS)). Studies in each language were screened using the following inclusion criteria: (1) in vitro or in vivo experiments, (2) effects at P-gp mediated MDR in colorectal cancer, and (3) subjects used as herbal medicines. In addition, the reference lists of potentially eligible articles were searched manually to identify additional relevant studies. An initial assessment was made by reading abstracts using the inclusion criteria. Then, articles meeting the inclusion criteria were read in full and we extracted data, including scientific name, herbal constituents, anticancer drug used, and pharmacological outcomes.

## 3. Results

**Figure 1:**
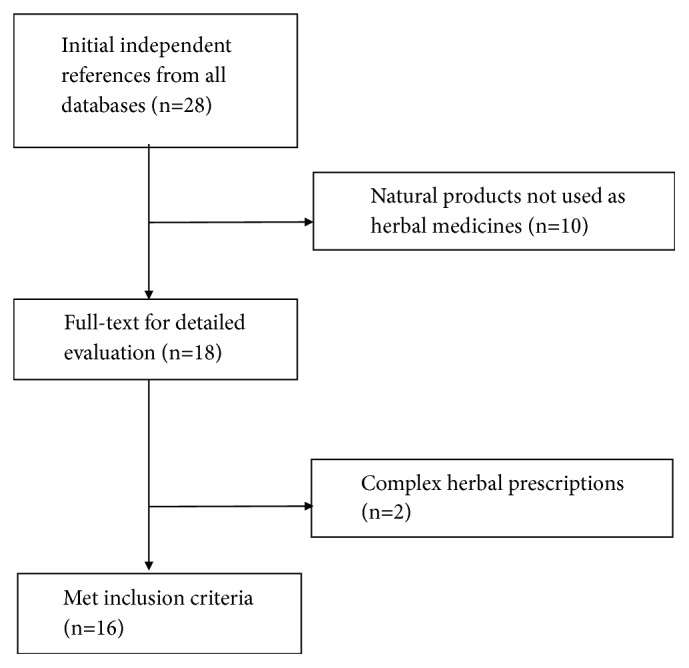
Scheme of the data selection process.

Our literature searches revealed potentially 28 relevant articles, of which 16 met our inclusion criteria (Figure 1). 16 studies were conducted in vivo, and two studies using* C. longa *and* H. Diffusa *were both conducted in vivo and vitro. To examine the intestinal drug absorption in CRC, 7 studies used Caco-2 cells, 3 used HCT-8 cells, 2 used HCT-15 cells, and 1 each used THC- 8307, SW480 cell, and rat jejunum membranes. One study used LoVo/ADR, HCT116/L, and Caco-2/ADR cell lines.

Of the 16 studies, 3 studies were conducted using different herbal constituents extracted from* Salvia miltiorrhiza *and 2 studies were designed to evaluate curcumin with different ways. Finally, a total of 14 kinds of herbal medicines—*Salvia miltiorrhiza, Curcuma longa, Sinomenium acutum, Stephania tetrandra, Bufo gargarizans, Coptis japonica, Piper nigrum *and* Piper longum, Hedyotis diffusa, Schisandra chinensis, Glycyrrhiza glabra, Glycyrrhiza inflate, Daphne genkwa, Stemona tuberosa *Lour*, Andrographis paniculata*—can be credited with the potential to reverse P-gp-mediated multidrug resistance in specific drug-resistant CRCs. All herbal constituents, including their pharmacological effects observed experimentally, are briefly described below and summarized in Table 1.

### 3.1. *S. miltiorrhiza* Radix

The radix of* S. miltiorrhiza* (Chinese name, Danshen) has been traditionally used for the prevention and treatment of cardiovascular diseases.* S. miltiorrhiza* extracts contain tanshinones (I, IIA, and IIB), cryptotanshinone, tanshinol (I and II), and salviol, among other compounds [19]. Respectively, Tanshinone IIA seemed to be the substrate of P-gp, considering the decreased transport of digoxin in P-gp overexpressing membrane, and low oral bioavailability is explained by the first-pass metabolism [20]. Tanshinone IIB showed the inhibition of uptake of digoxin and vinblastine in P-gp or MRP1 membrane vesicles [35]. Hu et al. evaluated the inhibitory effects of five tanshinones (tanshinone I, tanshinone IIA, cryptotanshinone, dihydrotanshinone, and miltirone) on doxorubicin (DOX) and irinotecan efflux in Caco-2 cells. They found that two tanshinones in particular, cryptotanshinone and dihydrotanshinone, downregulated P-gp mRNA and protein expression and inhibited P-gp ATPase activity, thereby increasing intracellular accumulation of P-gp-substrate anticancer drugs [21].

### 3.2. *C. longa* Rhizome

Curcumin, extracted from the rhizomes of* C. longa*, is well known as a major component of curry dishes in many Asian countries. Curcumin has been used not only for cooking but also in traditional medicine. It is known to be useful in various conditions, including cardiovascular health, cognitive function, and cancer treatment [36–38].

Curcumin has also been proposed as a supportive medicine in the chemotherapy of CRC. At concentrations greater than 25 *μ*M, curcumin was shown to enhance the sensitivity of human colon cells to treatment with vincristine, cisplatin, fluorouracil, and hydroxycamptothecin by suppressing the expression of the MDR gene and P-gp in vitro. The combination of curcumin and vincristine also significantly inhibited xenograft growth in vivo by reducing expression of MDR1 [22]. A subsequent study in 2010 testing extracts from rhizomes of* C. longa* and* Curcuma *spp. on Caco-2 cells showed increases in intracellular accumulation of the anticancer agent rhodamine 123 (R123) in both parental Caco-2 cells and vinblastine-selected Caco-2 cells. Moreover, these curcuminoids downregulated P-gp expression in drug-resistant Caco-2 cells, as evidence by a significant decrease in the efflux ratio of daunorubicin [23]. Tumerones, another class of sesquiterpenoids isolated from* C. longa*, were found to facilitate transport of curcumin into intestinal Caco-2 cells and enhance inhibition of P-gp activity, suggesting the potential of this agent to improve absorption of curcumin in the intestine [39].

### 3.3. *S. acutum* and* S. tetrandra* Rhizome

The rhizome of* S. acutum *and* Stephania tetrandra *(Chinese name, Fang ji) has been used as a folk remedy for rheumatic and arthritic diseases and neuralgia [40, 41]. In a recent study, sinomenine, extracted from the stem of* S. acutum*, was found to enhance the sensitivity of CRC cells to DOX [24]. Specifically, sinomenine at a concentration of 500*μ*M, which did not decrease cell viability alone, increased the sensitivity of DOX-resistant MDR-Cacp-2 cells to added DOX. This action was associated with downregulation of MDR1 and COX2 (cyclooxygenase 2). Consistent with this latter effect, treatment of MDR-Caco-2 cells with sinomenine decreased the release of prostaglandin E2 (PGE2), which was significantly elevated in these cells compared with parental Caco-2 cells. Furthermore, two bisbenzylisoquinoline alkaloids, tetrandrine and fangchinoline, extracted from* S. tetrandra* also showed to work as P-gp substrate in colon cancer cells. Choi et al. found that tetrandrine (3.0 microM) and fangchinoline (3.0 microM) increased the accumulation rates of rhodamine 123 in HCT cells whereas there was no effect on the cytotoxicity to P-gp negative cells [25].

### 3.4. *B. gargarizans*

Secretions from the skin and auricular glands of* B. gargarizans* (Asiatic toad) are called CahnSu in traditional Chinese medicine. Traditionally used against cardiac arrhythmias because of its cardiotonic effects [26], this secretion has recently been shown to have potential for cancer treatment. Bufadienolides—steroids isolated from the skin and parotid venom glands of the toad* B. gargarizans*—were shown to induce p53-mediated apoptosis in esophageal squamous cell carcinoma cells in vitro and in vivo [42]. In CRC cells, cinobufagin, another representative steroid, showed a reversal effect on P-gp-mediated MDR. Cinobufagin significantly enhanced the sensitivity of P-gp-overexpressing cells to DOX without affecting the corresponding parental cells. Studies of this agent further revealed that the mechanism of action involved noncompetitive inhibition of P-gp ATPase activity [26].

### 3.5. *C. japonica* Makino Rhizome

Haung Lian, the Chinese name for the rhizome of* C. japonica*, is widely used in various formulations for treating intestinal infection and inflammation; it is also known to possess central nervous system- (CNS-) depressant activities. The best known alkaloid in extracts of* C. japonica* is berberine, which has attracted considerable interest for its therapeutic potential in metabolic diseases [43]. More recently, Min et al. tested whether six components from* C. japonica *rhizome—6-([1,3]dioxolo[4,5-g]isoquinoline-5-carbonyl)-2,3-dimethoxy-benzoic acid methyl ester, oxyberberine, 8-oxo-epiberberine, 8-oxocoptisine, berberine, and palmatine—increased the sensitivity of five tumor cell lines—A549 (lung adenocarcinoma), SK-OV-3 (ovarian), SK-MEL-2 (skin melanoma), XF498 (CNS), and HCT15 (colon)—to paclitaxel. Among the compounds tested, 8-oxocoptisine showed significant inhibitory activity against P-gp-mediated MDR in HCT15 cells [27].

### 3.6. *P. nigrum* and* P. longum* Fructus

*P. nigrum* (black pepper) is a common spice and food additive. The fruit of* P. nigrum* and* P. longum* (Chinese name, Bi bo) has been used in traditional medicine for its important medicinal and preservative properties in intestinal diseases. It is also included in many traditional formulas to enhance the activity of other bioactive compounds, such as curcumin [44]. Its anti-inflammatory, antimicrobial, antioxidant, and anticarcinogenic activities have been experimentally verified [45]. In an attempt to identify derivatives that better interact with P-gp than piperine, Syed et al. designed the piperine analogs, Pip 1 and Pip 2, in silico and tested them in vitro. These latter experiments demonstrated that both analogs were better able to reverse the resistance to vincristine, colchicine, or paclitaxel in KB (cervical) and SW480 (colon) cancer cell lines than piperine. In support of these findings, these researchers further showed that Pip 1 and Pip 2 increased accumulation of the P-gp substrate R123 in these drug-resistant cells [28].

### 3.7. *H. diffusa* Herba

*H. diffusa* is a slender, annual plant that is widely distributed throughout Asia. The whole herb of* H. diffusa*, named Bai Hua She She Cao in Chinese, has traditionally been used to treat inflammation, urethral infection, contusions, and ulcers. Ye et al. found that an aqueous extract of* H. diffusa* exerted protective effects against renal inflammation in vivo [46]. In addition, an ethanol extract of* H. diffusa* was shown to increase the sensitivity of HCT-8/D-FU cells to 5-fluoruricil (5-FU) by inhibiting drug efflux and thereby increasing intracellular accumulation of 5-FU. Notably, this latter extract was shown to significantly downregulate mRNA and protein expression of drug efflux pumps, including ABCC1/MRP1 and ABCB1/P-gp [29].

### 3.8. *S. chinensis* Fructus

The fruit of* S. chinensis* is a medicinal herb whose Chinese name, Wu wei zi, means containing five flavors: salty, sweet, sour, spicy, and bitter. The active components of* S. chinensis *include deoxyschisandrin, *γ*-schisandrin, schizandrin, and gomisin A [47]. Numerous investigations of the effects of* S. chinensis* on hepatic damage have been conducted in recent decades [48, 49]. A comparison of the effects of* S. chinensis *extract and its lignin components gomisin A, gomisin N, and schizandrin C in Caco-2 cell lines showed that the transport of gomisin N is mediated by the MRP transporter. This indicates that gomisin N may bind competitively to MRP and P-gp [50]. In addition, *γ*-schizandrin was subsequently shown to exert an MDR-reversal effect in THC-8307/OXA human colon carcinoma cells by decreasing the expression of P-gp [30].

### 3.9. *G. glabra*,* G. inflate* Rhizome, and* D. genkwa* Flos

The root of* G. glabra* and* G. inflate* (Chinese name, Gancao) would be the most popular herbal medicine involved in approximately over 70% of herbal complex formulas. It was widely used for the bronchial remedies, gastrointestinal remedies, and mollification of toxicity traditionally. One of the main flavonoid extracted from* G. glabra* is glabridin, exhibiting cytotoxic activity, antimicrobial activity, etc. [51]. Cao et al. found that glabridin is a substrate for P-gp from the result that glabridin (2.56 microM) inhibited P-gp mediated transport of digoxin, but stimulating P-gp/MDR 1 ATPase activity. In addition, the systemic bioavailability of glabridin increased when combined with verapamil [31]. The oral administration of* G. inflate* was also found to inhibit P-gp in the intestinal membrane of rats slightly. In the same study,* D. genkwa* (Chinese name, Yan hua) decoction showed stronger P-gp inhibition effect than* G. inflate *and it could also decrease the permeability of secretory transport of rhodamine 123 across the jejunum tissues. Furthermore, combination of G. inflate and* D. genkwa* more significantly inhibited P-gp function compared to single administration of each herb [32].

### 3.10. *S. tuberosa* Lour Rhizome

Bai bu gen, the root of* Stemonaceae* family, was used in the treatment of respiratory diseases and the anthelminthic for a thousand years, and several alkaloids from S. tuberosa were verified for their antitussive activity in 2003. [52] Neotuberostemonine and neostenine, two major alkaloids, were P-gp substrates with exhibiting good intestinal absorptions, which means it can be orally active components. In addition, verapamil and cyclosporine A, P-gp inhibitors, resulted in decrease in secretory transport of two alkaloids and enhancement of absorptive transport of both alkaloids [33].

### 3.11. *A. paniculata* Herba

*A. paniculata *is a traditional herb common in Southeast Asia and found from India to China. It is known for the hypotensive effect and used for symptomatic relief of respiratory tract infections [53, 54]. Along with traditional usage, Ajaya et al. found that the methanolic extract of* A. paniculata*, especially dichloromethane fraction, exhibited anticancer and immunomodulatory activities by inhibiting proliferation of HT-29(colon cancer cell) and proliferating human peripheral blood lymphocytes [55]. Andrographolide, another major component extracted from* A. paniculata*, might modulate MDR by downregulating the overexpression of P-170 on HCT-8/5-FU cell line. The low concentration of andrographolide has no significant effectiveness, whereas it appeared to be effective when coadministered with 5-FU, Adriamycin, and cisplatin [34]. It implies the possibility of* A. paniculata *as chemosensitizer with anticancer drugs in colon cancer.

## 4. Discussion

MDR is a major obstacle that severely limits the efficacy of clinical chemotherapy, especially in the treatment of CRC. Among several mechanisms related to MDR, P-gp mediated MDR takes a significant part pathologically. The brief mechanism of P-gp mediated MDR is as follows: the mechanism of drug efflux reflects the structural transition of P-gp, which starts with dimerization of the NBDs to form an ATP-binding pocket. This allows the binding of compounds to P-gp. Upon binding of the substrate to the high-affinity drug-binding sites formed by 12 TMHs in the internal surface, the TMDs undergo a conformational change, switching from an inward to outward orientation in association with efflux of the substrate to the extracellular environment. ATP hydrolysis provides the energy for the dissociation of NBDs and resetting of the initial P-gp conformation [56]. Notably, most drugs used in chemotherapy for CRC, including targeted agents, are substrates of P-gp and thus show diminished intracellular accumulation [2, 57].

Since the first effort in 1981 by Tsuruo et al. [58], many attempts have been made to identify effective P-gp inhibitors. First-generation inhibitors were originally developed for other indications but were discovered to inhibit P-gp. Other examples of early-discovered inhibitors include immunosuppressants such as cyclosporin A; the antihypertensives, reserpine, quinidine and yohimbine; and antiestrogens, including tamoxifen and toremifene [9]. Many first-generation compounds are themselves substrates for P-gp and compete with coadministered substrates for efflux. As a result, high doses of these agents were required to have an impact on CRC. Because of their high cellular toxicity and nonselective effects, their use has been limited and led to the development of second-generation compounds [59]. To compensate for the defects of first-generation agents, which have low potency and specificity and high cellular toxicity, researchers structurally modified first-generation inhibitors, yielding second-generation compounds. Representative second-generation inhibitors include compounds such as PSC 833, a nonimmunosuppressive analog of cyclosporin A; dexverapamil, the R-isomer of verapamil lacking cardiac effects; and biricodar, also known as VX-710 [59, 60]. Despite improved performance, these agents could still not be used clinically, at least in part because most second-generation inhibitors also inhibited cytochrome 3A4 (CYP3A4). This undesirable action reflects the competition between anticancer agents and MDR modulators for CYP3A4 activity, which led to unpredictable interactions with anticancer drugs and reduced the bioavailable drug to subtoxic levels [61]. Third-generation inhibitors were designed to be greater than 200-fold more potent than first- and second-generation compounds and show almost no pharmacological interactions with other drugs. Third-generation compounds such as tariquidar (XR9576), zosuquidar (LY335979), laniquidar (R101933), and elacridar (F12091) are currently awaiting clinical trials as P-gp inhibitors [62].

After the repeated clinical failures of the established resistance-modifying agents, researches seeking P-gp inhibitors from the natural products had the limelight. In this study, we reviewed 16 studies demonstrating 14 herbal medicines—*S. miltiorrhiza, C. longa, S. acutum*,* S. tetrandra, B. gargarizans, C. japonica, P. nigrum *and* P. longum, H. diffusa, S. chinensis, G. glabra, G. inflate and D. genkwa, S. tuberosa *Lour, and* A. paniculata*— that have been demonstrated to alter P-gp-mediated drug efflux in CRC. Most of these studies were conducted in vitro; only two studies using* C. longa *and* H. Diffusa *were conducted in vivo, but none were performed in humans. Among the reviewed studies, the most frequently used CRC cellular line was Caco-2 cell. As it develops microvilli on the apical surface and expresses intestinal transporters such as P-gp, it has been widely used model for study of transport characteristics of drugs from introduction in the early 1990s. [63, 64] These 14 herbal medicines showed clear effects against CRC cells that were resistant to specific anticancer drugs, including doxorubicin, daunorubicin, vinblastine, vincristine, paclitaxel, verapamil, 5-FU, and oxaliplatin. The mechanism of herbal medicines against MDR mediated by P-gp can be summarized into two, acting as a P-gp substrate or downregulating P-gp expression in cellular membranes.

The limitation of our review pertains to the paucity of data. Further investigations in a clinical setting and more detailed mechanistic studies are warranted. Also, the fact that agents that modulate MDR may not act by directly inhibiting P-gp should also be taken into account. Our decision to exclude natural products, not used as herbal medicine, might also be criticized. However, we strongly feel that rather than listing a vast store of natural plants, proposing the practical herbal medicines could give a cue to further progress.

Above all, we observantly investigated natural products which were used as traditional medicine in East Asia. Since these herbal medicines have been used for thousands of years until nowadays, there are large amount of accumulated information of each products. An advantage of herbal medicine is that they function as multicomponent to multitarget (MCMT) agents. Thus, the overall potency/efficacy of herbal medicines cannot be defined by the action of a single compound derived from the herb. In addition to their MCMT mechanism, herbal medicines are expected to exhibit low side effects, good tolerability, and additional anticancer pharmacological actions. These properties may allow herbal medicine to be used as an effective adjunct to chemotherapy in CRC.

## 5. Conclusions

In this review, fourteen natural products that have been used in traditional medicine everlastingly were highlighted as potential P-gp reversal agents in CRC. It is expected that additional investigations of natural herbs will establish a crucial role for these agents in the successful chemotherapy of CRC.

## Figures and Tables

**Table 1:** Summary of herbal medicines as P-gp reversal agents in CRC.

**Scientific name**	**Herbal constituents**	**Anti-cancer drug**	**Model**	**Pharmacological outcome**	**Reference**
***Salvia miltiorrhiza***	Tanshinone IIA	Digoxin	Caco-2 cells, Rats	Tanshinone IIA inhibited digoxin in P-gp-overexpressing membrane vesicles with an IC (50) of 2.6 microM. P-gp-mediated efflux of TSA into the gut lumen	[19]
	Tanshinone IIB	Digoxin, Vinblastine	CaCo-2 intestinal cells, Rats	At the free plasma concentrations of Tashinone IIB in the range of IC50 values (28.1–37.3 mM), Tashinone IIB effectively inhibited digoxin and vinblastine transport.	[20]
	Tanshinone I, tanshinone IIA, cryptotanshinone, dihydrotanshinone miltirone	Doxorubicin	Caco-2 cells	Cryptotanshinone and dihydrotanshinone increased intracellular accumulation of the P-gp substrate anti-cancer drugs, presumably by downregulating P-gp mRNA and protein levels, and inhibiting P-gp ATPase activity.	[21]

***Curcuma longa***	Curcumin	Vincristine	HCT-8/VCR in vitro and in vivo	The sensitivity of cells to vincristine, cisplatin, fluorouracil, and hydroxycamptothecin was enhanced by suppressing the expression of the MDR gene and P-gp. The combination of curcumin and VCR in vivo significantly inhibited xenograft growth.	[22]
	Curcumin	Daunorubicin	Caco-2 cells	Decrease in the efflux ratio of daunorubicin by curcuminoids indicates that curcuminoids could modulate the efflux transporters expressed in Caco-2, especially P-gp.	[23]

***Sinomenium acutum***	Sinomenine	Doxorubicin	MDR-Caco-2 cells	Sinomenine downregulated P-gp expression in MDR-Caco-2 cells and enhanced the sensitivity of MDR-Caco-2 cells towards doxorubicin	[24]

***Stephania tetrandra***	Tetrandine, fanchinoline	Paclitaxel, Verapamil	P-gp positive HCT15 colon cancer cells	Tetrandine (3.0 microM) and fanchinoline (3.0 microM) enhanced the accumulation of rhodamine 123 in HCT15 cells. Both only affected the accumulation and residual rate of rhodamine 123 in P-gp-positive HCT 15 cells.	[25]

***Bufo gargarizans***	Cinobufagin	Doxorubicin	P-gp overexpressing LoVo/ADR, HCT116/L, Caco-2/ADR cells	Cinobufagin significantly enhanced the sensitivity of P-gp-overexpressing cells to DOX without affecting the corresponding parental cells.	[26]

***Coptis japonica***	6-([1,3]dioxolo[4,5-g]isoquinoline-5-carbonyl)-2,3-dimethoxy-benzoic acid methyl ester, oxyberberine, 8-oxo-epiberberine, 8-oxocoptisine, berberine, palmatine	Paclitaxel	five tumor cell lines in vitro; A549 (nonsmall cell lung adenocarcinoma), SK-OV-3 (ovarian), SK-MEL-2 (skin melanoma), XF498 (CNS) and HCT15 (colon).	8-oxocoptisine was of significant P-gp MDR inhibition activity with ED50 value 0.0005 pg/mL in HCT15 cell.	[27]

***Piper nigrum, Piper*** ***longum***	Piperine, Two small piperine analogs(Pip1,2)	Vincristine, Colchicine, Paclitaxel	Human KB 3–1, KB ChR 8–5, SW480 and HEK 293 cells	Both analogs, co-administered with vincristine, colchicine or paclitaxel were able to reverse the resistance in SW780 (CRC). Accumulation of P-gp substrate, rhodamine 123, in the resistant cells was observed as a result of alteration of the P-gp efflux.	[28]

***Hedyotis Diffusa***	Standardized ethanol extract *Hedyotis Diffusa *(EEHDW)	5-FU, Adriamycin	(i) In vitro: HCT-8 cells (ii) In vivo: male BALB/c nude mice injected with HCT-8/5-FU cells, HCT-8 cells	EEHDW or combination treatment could markedly downregulate the mRNA and protein expression of ABCC1/MRP1, ABCB1/P-gp, ABCG2/BCRP, Cyclin D1, CDK4 and Bcl-2 and upregulate the mRNA and protein expression of p21 and Bax in a dose-dependent manner in in xenograft tumors.	[29]

***Schisandra chinensis***	*γ*-Schisandrin compound	Oxaliplatin	MDR of human carcinoma of colon cell THC-8307/OXA	*γ*-Schisandrin decreased the expression of P-gp in THC-8307/OXA human colon carcinoma cells.	[30]

***Glycyrrhiza glabra***	Glabridin	Verapamil	CaCo-2 cells, MDCKII cells, rats	Glabridin is a substrate for P-gp. Low oral bioavailability is due to PgP/MDR1-mediated efflux and first-pass metabolism.	[31]

***Glycyrrhiza inflate***	Oral administration of decoction	Rhodamine 123	rat jejunum membranes in vitro	*G. inflate *showed slight inhibition of P-gp function in the intestinal membrane.	[32]

***Daphne genkwa***	Oral administration of decoction	Rhodamine 123	rat jejunum membranes in vitro	*D. genkwa* may be a strong inhibitor of P-gp compared to *G. inflate.*	[32]

***Stemona tuberosa *Lour**	Neotuberostemonine, Neostenine	Rhodamine 123	Caco-2 monolayer model	Both alkaloids were identified to be the substrates of P-gp.	[33]

***Andrographis paniculata***	Andrographolid (AG)	5-FU, Adriamycin, Cisplatin	HCT-8/5-FU multidrug-resistant colorectal cancer cell line	The reversal modulation of MDR by AG is related to its downregulation of overexpression of P-170. AG might act as a chemosensitizer when co-administered with anticancer drugs.	[34]
